# Flavivirus NS1 and Its Potential in Vaccine Development

**DOI:** 10.3390/vaccines9060622

**Published:** 2021-06-09

**Authors:** Kassandra L. Carpio, Alan D. T. Barrett

**Affiliations:** 1 Department of Biochemistry and Molecular Biology, University of Texas Medical Branch, Galveston, TX 77555, USA; klcarpio@utmb.edu; 2 Department of Pathology, University of Texas Medical Branch, Galveston, TX 77555, USA; 3 Sealy Institute for Vaccine Sciences, University of Texas Medical Branch, Galveston, TX 77555, USA

**Keywords:** flavivirus, NS1, vaccine

## Abstract

The Flavivirus genus contains many important human pathogens, including dengue, Japanese encephalitis (JE), tick-borne encephalitis (TBE), West Nile (WN), yellow fever (YF) and Zika (ZIK) viruses. While there are effective vaccines for a few flavivirus diseases (JE, TBE and YF), the majority do not have vaccines, including WN and ZIK. The flavivirus nonstructural 1 (NS1) protein has an unusual structure–function because it is glycosylated and forms different structures to facilitate different roles intracellularly and extracellularly, including roles in the replication complex, assisting in virus assembly, and complement antagonism. It also plays a role in protective immunity through antibody-mediated cellular cytotoxicity, and anti-NS1 antibodies elicit passive protection in animal models against a virus challenge. Historically, NS1 has been used as a diagnostic marker for the flavivirus infection due to its complement fixing properties and specificity. Its role in disease pathogenesis, and the strong humoral immune response resulting from infection, makes NS1 an excellent target for inclusion in candidate flavivirus vaccines.

## 1. Introduction

The Flavivirus genus contains many important human pathogens, including dengue (DEN), yellow fever (YF), Japanese encephalitis (JE), West Nile (WN), tick-borne encephalitis (TBE), and Zika viruses (ZIK) [1]. These viruses are commonly transmitted to vertebrate hosts via the bite of an infected mosquito or tick. Although the majority of infected humans are usually asymptomatic, those who have symptomatic disease manifest clinical symptoms that include fever, headache, and fatigue. A small percentage of individuals infected can progress to a severe disease state, including viscerotropic disease with YFV, neurotropic disease with encephalitic flaviviruses, and in the case of DEN, severe DEN, DEN hemorrhagic fever (DHF) or DEN shock syndrome (DSS) [2]. In rare cases, flavivirus infections can lead to fatal disease. While there are effective vaccines for humans to control JE, Kyasanur Forest disease, TBE and YF viruses, and promising live attenuated vaccines for dengue, there is an urgent need to develop vaccines for other flaviviruses, including WN and Zika [3,4,5].

## 2. Flavivirus Genome

The flavivirus genome is a single-stranded, positive-sense RNA approximately 11 kb in length that encodes three structural and seven nonstructural proteins. It is translated as a single polyprotein that is co- and post-translationally cleaved by viral and host proteases. The structural proteins include the capsid (C), pre-membrane/membrane (prM/M), and envelope (E) proteins found in the virion. The nonstructural (NS) proteins NS1, NS2A, NS2B, NS3, NS4A, NS4B, and NS5 form the viral replication complex (RC), and are mostly involved in viral RNA replication and dampening of the host innate immune response [6].

NS1 is an unusual protein for many reasons. Not only is it part of the replication complex, but is also involved in virus assembly, it is glycosylated, and exists in different multimeric forms intracellularly and extracellularly. As such, it has many properties that make it interesting in terms of flavivirus vaccine development.

## 3. NS1 Structures

**Figure 1:**
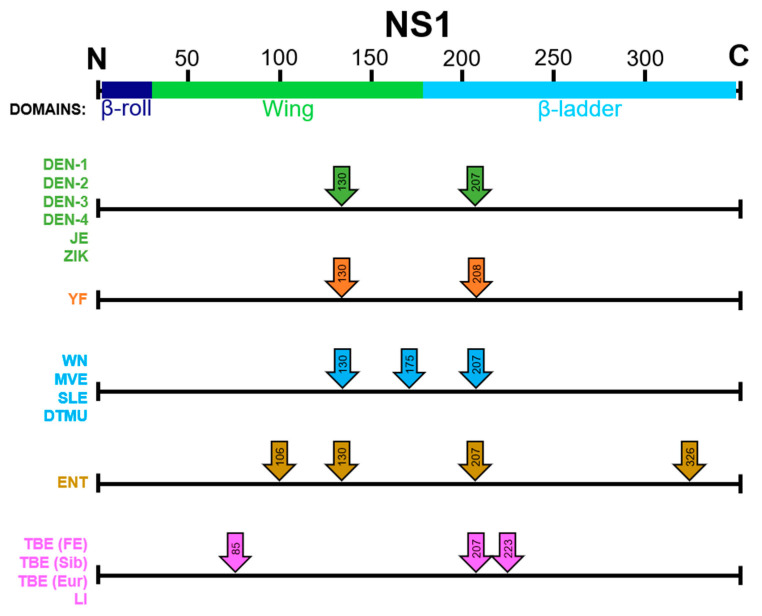
Location of putative or proven flavivirus N-linked glycosylation sites in the NS1 protein. DEN: dengue; JE: Japanese encephalitis; ZIK: Zika; YF: yellow fever; WN: West Nile; MVE: Murray Valley encephalitis; SLE: St. Louis encephalitis; ENT: Entebbe bat; TBE: tick-borne encephalitis; FE: Far East; Sib: Siberian; Eur: European; LI: louping ill; DTMU: duck Tembusu.

**Figure 2:**
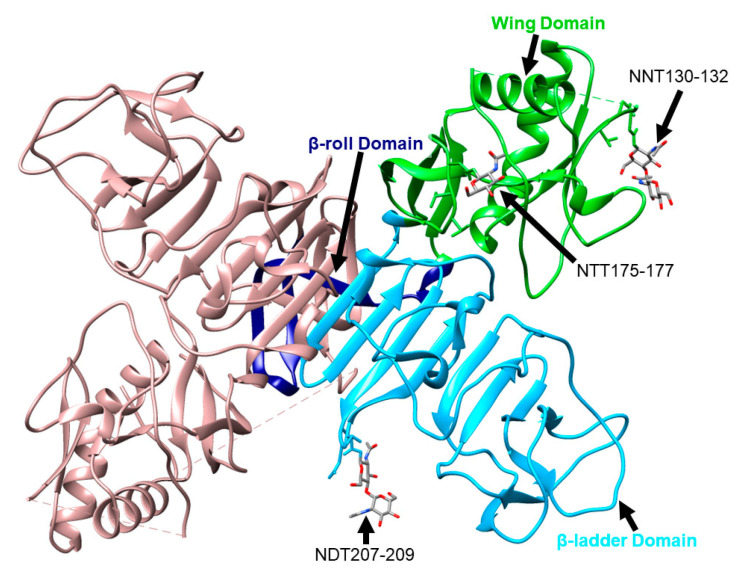
WNV NS1 dimer with one monomer colored in light pink and the second monomer colored as follows: β-roll (navy), wing domain (neon green), and β-ladder (light blue). The three glycosylation motifs of one monomer are included with their corresponding amino acids, residues, and N-linked glycans. This structure was colored using Chimera 1.14 and can be found at the PDB 4O6D.

NS1 is a highly conserved protein consisting of 352 amino acids with an approximate molecular weight of 40 to 50 kDa, depending on its glycosylation status (Figure 1). The majority of the Flavivirus genus members have two N-linked glycosylation sites at asparagine 130 and 207, including all four DEN serotypes, JE, and ZIK viruses; YF has glycosylation sites at the positions 130 and 208 [7]. A few members, such as WN, St. Louis encephalitis, and Murray Valley encephalitis (MVE) viruses, have a third glycosylation site found at amino acid position 175 [8]. Interestingly, the Entebbe bat virus (ENTV) has four potential N-linked glycosylation sites in NS1, including the two commonly found in all flaviviruses as well as at the residues 106 and 326 [9]. TBEV and louping ill (LI) have three putative N-linked glycosylation sites at the residues 85, 207, and 223 [10]. The amino acids at each glycosylation motif are characterized by N-X-T/S [11]. Intracellularly, NS1 initially exists as a monomer, but in the endoplasmic reticulum (ER) it forms a dimer due to the highly conserved cysteine residues present on the carboxy terminal, forming disulfide bonds [12,13]. Cryo-electron microscopy (EM) has identified the following three domains in each NS1 monomer: β-roll, wing, and β-ladder domains (Figure 2) [14]. The β-roll domain consists of amino acids 1–29 and contains two β-hairpins used in the dimerization of the protein. The amino acids 30–180 form the wing domain, which contains the potential Asn85, Asn106, Asn130, and Asn175 glycosylation sites. Finally, the β-ladder is formed by the C-terminal region of NS1, contains the potential Asn207, Asn208, Asn223, and Asn326 glycosylation sites, and each monomer contributes nine rungs to the anti-parallel ladder [15]. The wing domain and amino acids 296–335 have been found to be the most immunodominant in humans and mice [16]. Although NS1 lacks a transmembrane domain, once it reaches the cell surface, it exists as a hydrophobic dimer on the cell membrane [17]. It is secreted as a hexameric species formed by three dimeric subunits and is dependent on the glycosylation machinery, and consequently, the glycosylation status of the protein available in the cell [18]. This hexamer forms a hydrophobic lipoprotein particle due to its lipid-rich central channel, which is thought to contribute to endothelial dysfunction in dengue infection [19].

## 4. Cofactor in Viral Replication

Flaviviruses cause invaginations in the ER membrane to form vesicles that are used for RNA replication involving the seven NS proteins that form the RC. The colocalization of NS1 and double-stranded RNA to these structures suggests that NS1 plays an important role in viral replication [20]. NS1 has also been shown to bind to NS4A, which is an interaction important for replication [21]. A mutation at DEN2 NS4A-Y41F decreased the infectious virus production, which highlighted the importance of the interaction between NS1 and this tyrosine located in NS4A [22]. Additional evidence suggests that the NS1 present in the ER lumen can associate with NS4B to either directly or indirectly regulate viral replication and form the replication complex [21,23]. There are 12 cysteine residues in NS1 that form six disulfide bridges that have been shown to be critical for viral replication, as alanine substitutions impaired viral RNA replication and abolished viral multiplication [24]. The disulfide bonds are located at Cys4-Cys15, Cys55-Cys143, Cys179-Cys223, Cys280-Cys329, Cys291-Cys313, and Cys312-Cys316 [25]. A leucine substitution at the NS1 residue 250 in both Kunjin and MVE viruses suggests that dimerization is not necessary for virus replication; however, this did attenuate the viruses, and it is possible that undetectable levels of dimeric NS1 were present [26,27]. Interestingly, NS1 is one of the few NS genes that is able to be trans-complemented in a cell line stably expressing the replication complex [28].

## 5. Role of NS1 in Dengue Pathogenesis

As stated above, NS1 is unusual in that it is an NS protein that is secreted from cells. Since DEN is the most important flavivirus, it is not surprising that the role in pathogenesis has been most studied for DEN, where it has been demonstrated to contribute to the vascular leak associated with DEN pathogenesis. Specifically, NS1 binds to cells via heparan sulfate and is subsequently internalized by dynamin- or clatherin-dependent pathways. Internalization is required for endothelial barrier dysfunction, which is mediated by host proteases that disrupt the endothelial glycocalyx-like layer [29]. Anti-NS1 IgM and IgG from patients with DHF or DSS were found to be more reactive to endothelial cells than the sera from patients with DEN fever. These cross-reactive antibodies led to endothelial cell apoptosis and disease pathogenesis [30]. Macrophage migration inhibitor factor (MIF), an inflammatory cytokine, is correlated with DEN disease severity and is shown to contribute to NS1-induced endothelial autophagy, which in turn leads to vascular leakage [31]. This is upheld by the discovery that the level of NS1 in serum samples does not correlate with the extent of vascular leakage, and the levels of host factors, such as cytokines, the adhesion molecule ICAM-1, and deregulated phospholipid metabolites, are elevated in those with severe disease. However, it should be noted that the study involved the analysis of only 40 samples [32]. DENV is unique in that it can induce hyperpermeability in endothelial cells from different tissues [33].

The thrombocytopenia and hemorrhage seen in some cases of DEN infection can at least in part be attributed to the presence of circulating NS1 in the bloodstream. DENV NS1 has been shown to promote platelet aggregation, platelet adhesion to endothelial cells, and phagocytosis by macrophages through the binding of Toll-like receptor 4 (TLR4) [34,35], which can prevent clotting factors from controlling excessive bleeding. NS1 is also thought to interact with Beclin-1, an autophagy-related protein, to prevent apoptosis and increase viral replication in the early stages of infection. The elevated levels of caspases in the late infection stages degrade Beclin-1, leading to apoptosis [36]. Not surprisingly, the detection of circulating NS1 protein has been used as a diagnostic marker of acute DEN disease. However, a phase 2/3 study using the antiparasitic drug ivermectin found that the dose used in the study (400 µg/kg) was able to decrease the concentrations of NS1 in the plasma of DENV-infected patients, but did not affect the clinical outcomes [37]. Ivermectin was used because it was found to reduce the levels of circulating NS1 in DENV-infected individuals to undetectable levels compared to a placebo, but the results revealed that the two groups of patients had no difference in viremia clearance time nor a difference in the incidence of adverse events.

NS1 has a number of properties that make it a good immunogen to include in DEN vaccine candidates as it elicits a strong humoral response in DENV-infected individuals. In DEN infections, it can confer cross protection against all four serotypes, and NS1 antibodies have no role in antibody-dependent enhancement. However, the generation of anti-NS1 antibodies has the potential to lead to impaired host physiological function and tissue damage, because some anti-NS1 monoclonal antibodies (mAbs) have been shown to bind to host epitopes due to molecular mimicry [30]. Antibodies against DENV NS1 have been found in patient sera with cross-reactivity to human plasminogen [38]. This is hypothesized to enhance plasminogen activation, which could contribute to hyperfibrinolysis. A study in DENV2 immunized rabbits confirmed that anti-NS1 antibodies can inhibit thrombin activity and enhance plasminogen activation, which would impact blood coagulation and fibrinolysis [39]. It has been suggested that identifying and omitting the specific peptide sequences responsible for cross-reactivity could prevent these problems [40]. Subsequent studies using non-mouse adapted, mouse-virulent DENV2 strains (D2Y98P and EHIE2862Y15) found that secreted NS1 did not have a strong pathogenic role, nor did NS1 immunity confer protection in interferon αβ receptor knockout (A129) and interferon αβγ receptor knockout (AG129) mice. This is hypothesized to occur because the virulence of these strains relies on their structural components [41]. It is suggested that the role of NS1 in pathogenesis is strain-dependent, so NS1 may not be well suited as the universal vaccine immunogen for DENV.

## 6. Role of NS1 in Pathogenesis of Other Flaviviruses

The evidence from multiple flaviviruses suggests that NS1 may play a role in tissue tropism associated with a particular disease. In addition to the studies described on DEN above, WNV and JEV NS1 only interact with the glycocalyx on brain endothelial cells, and YFV NS1 has its strongest effect in liver endothelial cells [33]. ZIKV NS1 can lead to hyperpermeability in the umbilical vein and brain endothelial cells. Furthermore, ZIKV NS1 has been shown to alter the integrity of glycosaminoglycans on the extracellular matrix of trophoblast cells, and as a result disrupt the endothelial barrier function, which is thought to contribute to increased permeability of early gestation placentas [42]. Compared to the pre-epidemic Asian ZIKV strains, those isolated after 2012 seem to have a NS1-A188V mutation responsible, at least in part, for suppressing IFN-β induction by decreasing tumor necrosis factor receptor-associated factor (TRAF) family member-associated NF-κB activator (TANK)-binding kinase 1 (TBK1) phosphorylation [43]. In WNV, it has been shown that intracellular WNV NS1 can inhibit TLR3 transduction to prevent the IFN-β- and NF-кB-dependent promoters from activating an antiviral response [44].

## 7. Binding on Cell Surface

NS1 does not contain a transmembrane domain to facilitate membrane binding. Structural studies have identified an intertwined loop containing several hydrophobic amino acids found in the wing domain of NS1, which are thought to form a hydrophobic protrusion to bind the membrane [45,46]. In addition, NS1 associates with lipid rafts both during their formation in the Golgi apparatus and on the plasma membrane [47]. The surface expression and secretion of NS1 is regulated by the N-terminal amino acids 10 and 11, and the association is independent of any additional viral genes [48,49].

## 8. Anti-NS1 Antibodies and B Cell Epitopes

Prior to genome sequences and the identification of flavivirus-encoded proteins, NS1 was termed gp48 due to its mobility in protein gels. Anti-YFV live attenuated vaccine (LAV) 17D gp48 mAbs were found to confer protection to mice in passive transfer studies. The anti-gp48 mAbs with complement fixing (CF) activity were protective, while those without CF activity were not. Protection is thought to occur because of the antibody’s ability to promote complement-mediated cytolysis of 17D YF-infected cells [50]. In another study, the anti-NS1 mAbs 428, 423, 992, 917, 999, 979, 871, 925 (which are all YF-specific or YF- and Alfuy-specific) were thought to contribute to the protection of mice from intracranial 17D challenge through antibody-binding to virus-infected cells to prevent virus spread by recruiting T cells, macrophages, or complement to destroy the virus (Table 1). The antibodies may also attach to the antigens released when the virus particles are exiting the cell. These mechanisms may slow the virus from infecting nearby cells while a sufficient immune response is mounted [51].

In the case of MVEV, anti-NS1 antibodies from mice immunized with NS1 protein provided partial protection in F_1_, but not BALB/c mice from subsequent intracranial challenge. Interestingly, the sera from immunized mice did not activate complement-mediated cytolysis of infected cells, so an alternative pathway of cell lysis, such as antibody-dependent cellular cytotoxicity (ADCC), may contribute to virus clearance [52]. Six MVEV-specific, non-overlapping epitopes have been identified by MVEV NS1 mAbs. The M2-9A2 epitope was resistant to SDS denaturation and reduction, suggesting it is a linear epitope. However, the remaining epitopes were resistant to denaturation, but susceptible to reduction, which suggests they depend on disulfide bridge conformational changes [53].

It has been proposed that anti-DENV NS1 antibodies contribute to the pathogenesis of DEN through endothelial cell cross-reactivity, apoptosis induction, and inflammatory activation via a molecular mimicry mechanism. Antibody binding induces the lipid raft formation necessary for nitric oxide (NO) production, which leads to NO-mediated apoptosis [54]. The antibodies tested also induced NF-kB-regulated inflammatory activation, which leads to cytokine and chemokine production (IL-6, IL-8, and MCP-1) and ICAM-1 expression [55]. Structural studies using DENV NS1 revealed the protective mechanism of the mAb 2B7. The mAb interfered with the NS1 wing and β-ladder domains. The wing domain motif WWG (residues 115, 118, and 119) is suggested to be critical for the attachment of NS1 to cells, and the tip of the β-ladder is necessary for the downstream events required for mediating endothelial dysfunction [56].

Three WNV anti-NS1 IgG2a mAbs (10NS1, 14NS1 16NS1, and 17NS1) were also protective against WNV challenge in a passive transfer mouse study. The protection was identified as Fc-ɣ receptor- I- and/or IV-dependent. The epitopes recognized by these mAbs contribute to the binding of cell surface and intracellular forms of NS1 [57]. However, the specific amino acids in the epitopes recognized by the mAbs have not been identified, but in yeast display binding activity studies, 10NS1, 14NS1, 16NS1, and 17NS1 appeared to bind to NS1 amino acids 1–157 and 158–235, 236–352, 1–157, 1–157 and 236–352, respectively [58].

ZIKV is proposed to induce at least two types of mAbs that target NS1 based on their mechanism of action. An example of the first type is 4F10, which binds the C-terminal region, can trigger Fc-ɣ receptor-mediated phagocytosis, and inhibits ZIKV infection through effector cells. MAbs 3G2 and 4B8 are examples of the second type, which bind the N-terminal region and can inhibit infection through ADCC without effector cell assistance through both Fc-ɣ receptor-independent and -dependent mechanisms [59]. These results suggest that the protective efficacy of a mAb may be linked with its epitope recognition. Protection is hypothesized to occur through the inhibition of viral replication or the disruption of the production of infectious viral particles [59]. MAbs Z15 and Z17 were protective in non-pregnant mice, while mAbs Z17, ZIKV-292, and 749-A4 were protective in pregnant mice [60]. These mAbs map to epitopes in the wing domain and the C-terminal tip of the β-ladder of ZIKV NS1 on the cell surface. Protection likely occurs through their Fc-mediated effector functions and further suggests that protection by ZIKV anti-NS1 mAbs is linked to their ability to bind cell surface NS1. However, this study does not exclude the possibility that the mAbs may be antagonizing the secreted form of NS1, and it did not establish whether or not the mAbs protected from ZIKV-associated fetal demise [60].

A flavivirus cross-reactive mAb, 1G5.3, recognizes DENV, ZIKV, and WNV NS1 proteins and may have an application as a therapeutic antibody. The mAb was able to prevent DENV NS1-mediated cell barrier disruption in human microvascular endothelial cells, DENV and ZIKV NS1 mediated disruption in human umbilical vein and placental trophoblast endothelial cells, and WNV- and DENV-NS1 mediated disruption in human brain microvascular endothelial cells [61]. DENV-infected mice were treated with mAb 1G5.3 or a 1G5.3 Fab fragment two days post infection (dpi), and were found to have significantly lower levels of viremia, vascular leakage, and NS1 protein. Using a lethal mouse challenge model for DENV, 80% survival was observed when mAb 1G5.3 was administered 1 dpi, and less protection was observed when the Fab fragment was administered instead. Overall, the results suggest mAb 1G5.3 can directly inhibit NS1 pathogenesis as well as contribute to Fc-dependent mechanisms such as ADCC. The mAb was also somewhat protective in lethal challenge models using ZIKV and WNV in mice [61].

Several flavivirus NS1 epitopes have been identified through structural studies or biochemical methods, such as enzyme-linked immunosorbent assays (ELISAs) (see Table 1). Cryo-electron microscopy of WNV NS1 in a complex with a mouse anti-NS1 mAb 22NS1 (which was found to be protective in mice through an unknown mechanism, and recognizes the soluble hexamer and surface-associated dimer) revealed an epitope consisting of the loop face amino acids 172–352 [62]. The mAb 22NS1 was also able to bind the C-terminal region encompassing the same amino acids of JEV NS1 [63]. However, it is not known if this mAb is protective in JEV infection. MAb 2B8 recognizes an epitope at JEV NS1 amino acids 225–233 and protected 70% of mice against a lethal challenge [64]. Finally, WNV mAb 3C7 was found to bind to residues 895–901 [65].

A unique, linear mouse NS1 epitope was discovered for JEV that recognizes amino acids 145–152, which could prove useful as a diagnostic marker specific for JEV since this region is not conserved in other flaviviruses [66]. In addition, JEV-specific NS1 epitopes have been identified by the mAbs 1H6, 10F7, 8B5, 7G1, 7C11, 4E3, HA12, 3H11, 3G11, 4E8, 5C6, and 6D8 (see Table 1) [67]. The mAbs 4D1 and 1E8, which map to NS1 residues 925–934 and 227–232, respectively, have been identified to be common among the JEV serocomplex viruses [65,67].

The anti-DENV NS1 mAbs 33D2 and 19-5 have been shown to recognize the NS1 wing domain. Based on the epitopes recognized by these mAbs, a peptide was synthesized to immunize mice. The resulting mouse sera recognized all four DENV serotypes, while having less endothelial cross-reactivity, and induced the complement-dependent lysis of infected cells through possible interactions with surface-bound NS1 dimers [14]. The mAbs 2H5 and 4H1BC recognize the NS1 epitope at the residues 193–209 for both DENV2 and ZIKV. The same study also identified two mAbs that recognized DENV2, but not ZIKV NS1. These include mAb 4F6, which bound to the amino acids 25–38, and mAb 4H2, which recognized the residues 127–143 [68]. Similarly, a major epitope was identified between amino acids 221 and 266 using DENV2-infected patient sera that was also found to be cross-reactive with DENV1 and DENV3 [69]. The mAb 15F3-1 is specific for DENV1 and recognizes the epitope at the residues 110–117 [70]. Several epitopes specific to DENV2 have been identified by the mAbs DB6-1, DB12-3, and DB38-1 [71]. Additional mAbs used to map both DENV2 and DENV4 epitopes include DB41-2, 5H5.4, and 1G5.3. This study also identified an epitope around the amino acids 111–121, which is able to recognize all four serotypes of DENV [72].

There are limited data regarding epitopes on other members of the flavivirus genus. However, a major ZIKV NS1 epitope was mapped to residues 118–147, based on binding studies, using ZIKV-positive patient sera, while DENV sera did not cross-react with this epitope [73]. Two TBEV NS1 epitopes were discovered at the amino acids 1–33 and 269–333, and finally, a single B-cell epitope was identified for Tembusu virus at the residues 269–274 [74,75].

## 9. Complement and ADCC

Anti-NS1 antibodies do not have neutralizing activity; however, they do activate Fc-mediated effector functions, such as ADCC or antibody-dependent cellular phagocytosis (ADCP) used to clear virus-infected cells [76]. One of the known roles of NS1 is complement antagonism, which prevents the recruitment of leukocytes to the site of infection, prevents opsonins from binding viral particles, and keeps the membrane attack complex from forming to stop the lysis of virus-infected cells. The NS1 protein of DEN, WN, and YF viruses has been found to attenuate the classical and lectin pathway activation by binding C1s and C4 in a complex to promote the degradation of C4 to C4b. The binding of C1s is thought to prevent the enzyme from also cleaving C2 to form the C3 convertase, which is a necessary step for all complement activation pathways [77,78]. Interestingly, DSS patients were found to have low serum C3 concentrations, which is indicative of complement activation. This activation can lead to the hallmark symptoms of DSS, such as the release of a fragment with potential shock-producing activity, the release of histamine to increase vascular permeability and vasodilation, and platelet activation that leads to blood coagulation [79].

## 10. Serology Using Complement Fixation and Neutralization Assays

Historically, it has been known since the 1940s that flaviviruses have antigens that mediate CF [80]. Indeed, CF together with neutralization were used as serologic tools to investigate the relationships of different flaviviruses. Following the identification of flavivirus-encoded proteins, it was shown that neutralization was associated with the envelope [E] protein and CF with the NS1 protein. Serologically, NS1 antibodies tend to cross-react with viruses only in a particular antigenic complex and, unlike the E protein, do not have epitopes shared across different antigenic complexes or by all flaviviruses [81]. In humans infected with DENV, the soluble CF antigen is mostly found in convalescent-phase sera from secondary infections [82]. Interestingly, the YF live attenuated 17D vaccine does not induce CF antibodies, while the wild-type YF virus infection does [83]. The reason is not known, but it provides a simple serologic method to distinguish between vaccine and wild-type virus-induced immunity.

## 11. Importance of Adding Carbohydrates

Flaviviruses have the following three glycosylated proteins: prM, E, and NS1. NS1 is unusual in that it is a NS protein that is glycosylated (Figure 1). For those flaviviruses with two NS1 N-linked glycosylation sites, high-mannose-type glycans are added to both sites of the monomer in the ER and trimmed by α-glucosidase I and II. At this point, NS1 reaches chaperones to be properly folded and proceed downstream to the Golgi, where a complex glycan is attached to Asn-130 [84,85]. Several antiviral studies using compounds that block ER glucosidases have shown promising results in virus-infected animals to reduce mortality, viremia, and viral RNA levels [86,87,88,89,90]. A couple of caveats to these studies are that the majority are only tested in response to DENV infections, and the safety and efficacy has not been proven in humans. An iminosugar, celgosivir, has been shown to cause NS1 to misfold and accumulate in the ER [91]. However, a subsequent small phase I, proof-of-concept clinical trial (ClinicalTrials.gov number NCT01619969) suggested the treatment did not reduce the viral load nor fever burden in DEN patients [92]. While a similar drug, castanospermine, prevented mortality in a DENV-infected mouse model, but *in vitro* assays demonstrated that the treatment had no effect on either WNV or YFV infection [93]. Recent findings in mammalian cells identified the oligosaccharyltransferase (OST) complex as important for the propagation of flaviviruses. Knock-out (KO) studies revealed that the catalytic components (STT3A or STT3B), but not their catalytic activity, are required for efficient DENV replication. More specifically, activity of the catalytic subunit, magnesium transporter 1, is necessary in the ER for efficient synthesis, folding, or recruitment of NS proteins [94]. Although STT3A and STT3B have been implicated in the glycosylation of asparagine in the flavivirus glycosylation motif (N-X-S/T), NS1 glycosylation differences were not detected in DENV-infected OST KO cells.

## 12. Effects of Removing Glycosylation Sites

The importance of the N-linked glycosylation motifs has been highlighted over the years through the generation of glycosylation-null mutants. The expression of DENV or WNV NS1 in Sindbis virus allowed a comparison between the glycosylated and non-glycosylated NS1 proteins, and sought to answer whether or not the extra glycosylation site in WNV NS1 compared to DENV NS1 offers any advantages to the virus. A glycosylation-null mutant of WNV NS1 did not change the pattern of surface level expression. However, the mutant was found in the supernatant at very low levels compared to WT NS1, which suggests that glycosylation is necessary for correct secretion, but not cell surface expression [48,95]. Furthermore, the generation of an attenuated WNV NS1 130-132QQA/175A/207A mutant was found to replicate to lower titers compared to the parental strain, and NS1 accumulated in the ER, which could cause reduced early replication or malformed replication vesicle formation [96]. All of the results to date for multiple flaviviruses are consistent with the N-linked glycan at position 130 being critical for replication, stabilization of the hexamer, and interaction of NS1 with complement components, while the glycan at position 207 is thought to facilitate secretion and extracellular protein stability [97,98]. Additionally, mutants lacking the N-linked glycosylation site at residue 207, or those containing synonymous mutations at both sites, had similar phenotypic characteristics to the parental virus in cell culture [7]. It has been shown that ablation of the second glycosylation site in DENV2 can increase neurovirulence over six-fold in mice and shorten the mean survival time [99].

## 13. NS1 as a Vaccine Immunogen

As described above, NS1 has a number of properties that suggest it has applicability to flavivirus vaccine development. These include its potential as a candidate vaccine immunogen, either as a recombinant protein or expressed in virus vectors. In addition, mutated NS1 has been used in candidate live attenuated vaccines. These are described in detail below.

### 13.1. Subunit Vaccine Candidates

In early studies, purified NS1 protein was studied as a subunit vaccine candidate for many flaviviruses. Rhesus macaques were administered with the YF vaccine strain 17D NS1 protein via the intradermal (ID) or subcutaneous (SC) routes, with boosters at 3 and 7 weeks. Four of the five monkeys immunized with NS1 survived a lethal challenge, which was attributed to either CF antibodies to NS1 in at least two of the surviving monkeys or the contamination of the NS1 used for inoculation with E protein, which resulted in neutralizing antibodies in the other two surviving monkeys [100]. Similarly, mice immunized with purified NS1 given by the intraperitoneal (IP) route developed complete immunity to 17D challenge, with the correlate of protection being high CF antibody titers [50]. The immunization of mice by the IP route with DENV2 NS1 also provided protection against a DENV2 intracranial (IC) challenge; however, cross-protection was not observed against the other DEN serotypes, which was thought to occur because the mice did not have sufficient time to accumulate high enough levels of CF antibodies [101]. In contrast, DENV NS1 immunization in rabbits, given by the combined ID and SC routes, did appear to offer cross-protection. A bacterially expressed YFV NS1 fusion protein containing a fragment of NS1, including NS1 amino acids 22 to 130, offered some protection in mice; however, complete protection was not observed, possibly due to the portion of NS1 that was used or the short immunization schedule prior to the challenge [102]. A later study used a bacterially expressed fragment of DENV2 E and NS1 fused to a staphylococcal A protein as an adjuvant, given by the SC route, and found this was able to protect mice from an IC challenge as well as produce high levels of neutralizing antibodies, although the exact role of the staphylococcal A protein was unknown [103].

A DNA vaccine, consisting of a plasmid DNA encoding JEV NS1 given by the intramuscular (IM) route, was able to protect 90% of mice from a lethal challenge. However, inoculation of the plasmid vector alone protected 40% of mice, suggesting that the NS1 immunization might have a low protective efficacy [104]. More recent studies with DENV2 suggest that an NS1 DNA vaccine can elicit both humoral and cellular immune responses, including T-cell proliferation [105]. In a vaccination regimen including DNA plasmid priming, given via IM electroporation, and two NS1 protein boosts, also given by the IM route, passive transfer of the sera from immunized mice to naïve mice was found to protect from a lethal challenge. The serum from NS1-vaccinated mice was also shown to induce effector functions on ZIKV-infected cells [76]. Similarly, two doses of a plasmid vaccine with DENV2 NS1 fused to a t-PA signal, administered IM to BALB/c mice, protected the mice from an IC challenge with mouse-adapted DENV2 [106]. This study also investigated the effects of IP challenge of vaccinated mice with a non-adapted DENV2, and found that the vaccine reduced tissue injury resulting from the challenge. This plasmid used in these studies is unique in that it causes NS1 to be secreted as a dimer. Subunit vaccines provide an excellent starting point that requires further investigation to assess which additional viral components, e.g., E protein, are needed to provide complete protection from a lethal challenge. Finally, it was shown recently that the vaccination of BALB/c mice via the IM route with a ZIKV NS1 DNA vaccine, with boosting 2 weeks later, elicited high levels of anti-NS1 antibodies and a T-cell response. The mice were partially protected against viremia following a ZIKV challenge. When this vaccine was administered by the IM route to Ifnar^−/−^ (interferon-αβ receptor knockout) mice, with boosting two and five weeks later, four/five mice developed high levels of NS1-specific antibodies. However, all of the mice developed severe disease after a 10^5^ PFU ZIKV challenge [107]. Additional studies suggest an NS1 DNA vaccine can confer protection against ZIKV infection in BALB/c mice as long as NS1 is effectively secreted. This protection is mediated by T cells [108].

### 13.2. Vectored Vaccine Candidates

NS1 has also been included in several recombinant virus vaccines, which express NS1 that is indistinguishable from naturally occurring NS1 in terms of glycosylation, molecular weight, and sedimentation properties. In a study using DENV4 structural proteins plus the NS proteins NS1 and NS2A expressed in a recombinant vaccinia virus and given by the ID route, mice inoculated with the construct displayed a weak immune response. This was thought to occur because part of the 5′ noncoding region was not included, which possibly led to poor translation of the polyprotein [109]. In a recombinant vaccinia virus encoding YFV NS1, NS2A, and NS2B, given by the IP route, partial protection was observed in the mice following a lethal, IC challenge [110]. Authentic DENV4 NS1 and NS2A included with its flanking sequences in a recombinant vaccinia virus was able to protect 100% of mice from a challenge; however, many mice displayed clinical signs of morbidity. The ability of the mice to survive the challenge was attributed to serum NS1-specific antibodies, as the sera elicited passive protection in mice [111]. A JEV vaccinia recombinant containing the prM, E, NS1, and NS2A proteins, given by the IP route, protected mice from a lethal challenge and induced neutralizing antibodies, likely due to the E and M proteins forming extracellular particles [112].

In a non-replicative adenovirus E1 deletion mutant containing the cytomegalovirus immediate early promoter and TBEV NS1 (known as Rad51), the mice immunized by the IP route produced a strong humoral response and >50% mice were protected against a TBEV challenge [113]. Furthermore, additional mouse studies of Rad51 showed that it can offer cross-protection against different TBE subtypes, probably due to the conserved genetic sequence of NS1 [114]. The studies involving an attenuated, recombinant vesicular stomatitis virus (rVSV)-based vaccine, expressing ZIKV prM-E-NS1 with a defective methyltransferase in VSV, showed promising results in BALB/c and A129 mice. To determine if the rVSV-based vaccine was immunogenic and attenuated, a single intranasal immunization of the rVSV–prM-E-NS1 vaccine was administered and did not cause significant weight loss nor clinical signs of infection, suggesting that this vaccine was sufficiently attenuated compared to the parental rVSV while stimulating a strong antibody response. The inclusion of the ZIKV NS1 protein contributed to enhanced IFN-γ and inhibited TNF-α production by T helper cells. After re-exposure to the ZIKV E protein, T cells were able to proliferate and induce the Th2 response, as well as an enhanced Th17 response [115]. In contrast, intranasal inoculation of the rVSV vaccine expressing only NS1 induced high levels of anti-NS1 antibodies, a T-cell response, and partial protection against ZIKV viremia in BALB/c mice. The vaccine given by the IM route did not protect Ifnar1^-/-^ mice against a 10^5^ PFU ZIKV challenge, while a 10^3^ PFU challenge caused viremia and weight loss [107]. Alternatively, a ZIKV-NS1 vaccine was generated using a modified vaccinia Ankara (MVA) vector and was able to protect 100% of IM-vaccinated CD-1/ICR mice from IC challenge [116]. The suggested mechanism of protection involves both Fc-mediated anti-NS1 antibodies that may trigger cell death by CF, ADCC, and phagocytosis, and CD8+ T cells targeting NS1 epitopes.

In comparison, the studies utilizing baculovirus-expressed NS1 did not show promising results, as immunization did not protect animals from virus challenge. DENV2 NS1 lysates, produced in a baculovirus expression system, used in mouse immunization studies, and given by the IP route, yielded unique results. Only the female BALB/c mice were protected after virus challenge, which is presumed to be due to the genetic background and greater immune reactivity of females due to the effects of sex hormones [117]. Subsequent studies investigated Rhesus macaques immunized by either the combined SC and ID routes, or only the SC route. The macaques vaccinated with lysates from a baculovirus-expressed construct consisting of the DENV4 C, M, E, NS1, and NS2A proteins found that the majority of animals were not protected, as seven out of nine developed viremia after challenge depending on the dose [118]. Finally, the lysates from *S. frugiperda* cells infected with baculoviruses-expressing YFV NS1 were administered by the IP route and were able to delay infection in mice challenged IC with YFV. However, anti-NS1 antibodies with ADCC activity were not detected [119].

### 13.3. Live Attenuated Vaccines

Several live, attenuated vaccine candidates targeting the NS1 glycosylation motifs have been generated with results further highlighting the importance of these conserved sites. Reverse genetics has been used to study a WNV NS1 mutant as a vaccine candidate. This study demonstrated that alanine substitutions at each of the three N-linked glycosylation sites would induce an attenuated neuroinvasiveness phenotype in mice, but was not stable enough to attenuate the virus for neurovirulence in all vaccinated mice due to reversion in vivo. However, the studies showed that all three glycosylation sites play a role in mouse virulence [8]. This could be due to the importance of the complex glycan attached to the asparagine at NS1 position 130, since this was the site of reversion in the virus collected from the animals that succumbed to the infection. To prevent reversion to virulence, additional substitutions at all three residues of the NS1 130–132 glycosylation motif, as well as the asparagines at positions 175 and 207, were introduced. The addition of mutations at the first glycosylation motif in NS1 130–132QQA/175A/207A did not significantly change the neurovirulence nor neuroinvasiveness compared to the alanine mutant NS1 130A/175A/207A, since the 50% protective doses from a lethal NY99 challenge were 50 and 80 PFU, respectively, which is not significantly different [120]. Similar studies have been carried out using YF and DEN2 viruses, with results in DENV2 differing from YFV and WNV. YFV 17D mutants with non-coding mutations at both glycosylation sites, or missing the second NS1 glycosylation site, had similar properties to the parental virus in cell culture, while the mutants lacking the first or both glycosylation sites attenuated the virus for mouse neurovirulence, reduced viral RNA accumulation and NS1 secretion, and delayed cytopathic effects (CPE) in SW-13 cells [7]. In contrast, the DENV2 mutant deglycosylated at the second N-linked glycosylation site had reduced CPE in LLC-MK_2_ cells, reduced NS1 secretion, and was attenuated for mouse neurovirulence [121].

The addition of the JEV NS1 gene into a chimeric single-cycle JE vaccine candidate TripliVAX JEV was found to be more protective in a mouse model than the inclusion of only JE prM and E genes, with NS1-5 originating from WNV. A small dose of 6.25 × 10^2^ infectious units was able to elicit neutralizing antibodies and provide protection from infection. This may be due in part to the ability of NS1 to enhance the proper maturation or release of E protein from virus-infected cells [122].

The DENVax-based vaccines were generated from an infectious cDNA clone based on the candidate live attenuated DENV2 strain 16681 following 53 passages in primary dog kidney cells (PDK-53), where each DEN serotype component was generated by replacing the prM and E genes with those from the serotype of choice. Interestingly, the mutations responsible for attenuation of the DENV2 PDK53 backbone are found in the 5′ non-coding region, NS1, and NS3 [123]. The NS1 mutation is found at residue 53 and is highly conserved among the DENV serogroup, where there is a glycine to asparagine substitution. It is thought to contribute to the attenuation of neurovirulence in mice, temperature sensitivity, and small plaque phenotypes [124]. The NS1-G53D substitution in PDK53 was also found to contribute to the decreased viral replication rates and attenuation of infection in *Ae. aegypti* mosquitoes, as well as the more rapid induction of type-1 IFN. The suggested mechanism of attenuation involves the misfolded NS1 interfering with the reticulum-resident host ribophorin 1 protein, which then leads to improper NS1 glycosylation. This is ultimately thought to activate the unfolded protein response as a result of ER stress and protein accumulation in the ER lumen [125]. Interestingly, in a different study, the mutation of DENV2 at NS1-T164S resulted in greater disease severity in mice [126].

A live attenuated JE-ChimeriVax vaccine has been constructed, consisting of the YFV 17D genome with the prM and E proteins replaced with the homologous JE live attenuated SA14-14-2 vaccine strain proteins (termed JE-CVax). A recent study showed that the JE-CVax vaccine given by the IP route was able to offer protection from lethal JEV SA14-14-2 and YFV 17D IC challenges in AG129 mice [127]. However, no anti-17D neutralizing antibodies were detected, suggesting that YF protection could be due to anti-NS1 antibodies. It is not known if this is also true for humans, but it opens the door to testing other chimeric vaccines to see if they also confer cross-protection.

A candidate ZIKV LAV containing substitutions at the E glycosylation site (N154A) and both NS1 glycosylation sites (N130A and N207A), given by the SC route, was able to protect mice from a lethal WT challenge. The E protein mutation alone attenuated the pathogenicity and neuroinvasion of the virus [128]. A study looking at the effects of a double ZIKV NS1 glycosylation mutant (NS1- N130Q + S132A + N207Q + T209V) found that the immunization of female mice, given by the SC route prior to pregnancy, conferred protection and resulted in lower levels of viral RNA recovered from the dam spleen and brain, as well as in the placenta and fetal heads compared to placebo-immunized mice. Importantly, infectious virus was not recovered from the placenta or fetal heads from dams immunized with the candidate LAV [129]. The mice inoculated with the mutant with substitutions in both E and NS1 were found to have no or mild pathology in tissues, and elicited strong humoral and cellular immune responses. Serial passaging of the virus revealed that the genome was stable, and the mutant could not replicate as well as wild-type ZIKV in cell culture [130]. Additionally, the passive transfer of sera from the mutant-infected mice also protected the A129 mice from WT ZIKV infection. Similar to the studies described above, the removal of the NS1 glycosylation sites shows promising results in a candidate LAV for ZIK.

There are two licensed live attenuated flavivirus vaccines in wide use today with mutations in their NS1 protein: YF 17D and JE SA14-14-2. The YF vaccine strain 17D was derived by serially passaging the WT Asibi strain in chicken tissue, and it differs from its parental strain by 20 amino acid substitutions. While eight of the substitutions were found in the E protein, interestingly, one of the amino acid substitutions from Asibi to 17D lies within the NS1 region at position 307 [131,132]. It is not known if this substitution contributes to attenuation. However, the high-fidelity replication complex in 17D appears to play a major role in viral attenuation [133]. Similarly, the JE vaccine strain SA14-14-2 has one nucleotide change in NS1 compared to its virulent parent strain SA14, but it does not encode an amino acid substitution and it is unknown if this contributes to attenuation [134]. However, SA14-5-3 differs from SA14-14-2 at NS1-351, which may play a role in the reduced immunogenicity of SA14-5-3 [135].

### 13.4. Trans Complementation as a Vaccine Strategy

The need for a new vaccine strategy is important for many flaviviruses of public health concern that are highly pathogenic, especially for elderly and immunocompromised individuals at high risk for infection. A replication defective WNV vaccine, with deletion of NS1 (WNV-ΔNS1), was propagated in Vero cells expressing the WT NS1 protein to mean peak titers of 1 × 10^8^ IU/mL, and it was not found to recombine with the WT NS1 sequence of NS1-expressing Vero cells after 15 passages. When WNV-ΔNS1 was inoculated by the IP route in type I IFN receptor-deficient mice, neither morbidity nor mortality were observed. Immunocompetent C57BL/6 mice were vaccinated with either one or two doses. Both experiments induced high neutralizing antibody titers and protected the mice from a lethal challenge of WT WNV [136].

A JEV vaccine using the same NS1 *trans*-complementation platform as WNV was generated. The replication-defective JEV with the NS1 deletion (JEV- ΔNS1) also replicated to high titers using BHK-21 cells expressing WNV WT NS1 and retained its 295-residue deletion over 15 passages. The C57BL/6 mice were inoculated by the IP route with multiple dilutions of JEV- ΔNS1, in which all mice survived with no signs of morbidity. The ICR mice were IC inoculated to test neurovirulence and they were compared to mice inoculated with WT JEV. All of the mice inoculated with WT JEV died within 10 days of inoculation, whereas all of the mice inoculated with JEV- ΔNS1 survived without neurological signs. A single immunization was able to confer protection from WT JEV equivalent to the licensed JEV SA14-14-2 vaccine and provided dose-dependent protection against infection from WT WNV [137]. These studies identify a novel vaccine platform that shows promising results for effective flavivirus vaccine development.

## 14. Conclusions

NS1 is a multi-functional protein playing roles in virus replication, virus pathogenesis and the immune response to virus infection (Table 2). Currently, there are only vaccines for a few flavivirus diseases, and the methods used to generate these vaccines cannot be applied to every flavivirus. Due to its role as a key flavivirus virulence determinant and contribution to protective immunity, NS1 provides an excellent option to explore when considering which genes to include in a candidate flavivirus vaccine. Unfortunately, the early candidate subunit and baculovirus vectored vaccines, including NS1, did not show the level of protective efficacy that would be required from a licensed vaccine. However, the licensed LAVs and some of the other vectored vaccines provide a proof-of-concept for successful vaccine candidates. The studies involving NS1 glycosylation knockouts highlight the importance of carbohydrates to correct the function of the protein and its relevance in the future generation of flavivirus vaccines. It is clear that generating a vaccine including NS1 for DENV presents a potential unique solution to the antibody-dependent enhancement dilemma associated with severe disease, which is related to antibodies against E protein epitopes. However, it should be remembered that current flavivirus diagnostics focus on NS1 antibodies to provide an accurate identification of a particular virus infection due to flavivirus cross-reactive antibodies induced by the E protein, which complicate flavivirus diagnosis and identification. Since NS1 is not located in the virion, the immune response does not produce neutralizing antibodies to NS1, but rather ADCC and CF antibodies, which nonetheless mediate a protective immune response. However, the early DEN studies undertaken remind us that animal models do not necessarily recapitulate human disease, and experiments utilizing mice and monkeys resulted in very different outcomes. This may be in part due to the use of immunocompetent mice to establish protection against neurotropic disease, whereas A129 and AG129 mice that succumb to dengue-type disease are used as the mouse model of choice today. This is because A129 mice lack the receptor for IFN-α/β, but retain their IFN-ɣ receptor, which allows for the reduction in viral replication during early infection and the clearance of the virus specifically by CD8+ T cells. The AG129 mice lack both types of interferon receptors and are therefore useful for evaluating antibody responses that might not be present in mice with intact receptors due to the help of cellular immunity [138]. Overall, the inclusion of NS1 in a majority of vaccine platforms has shown promising results and highlights the importance of this protein and its glycosylation status in flavivirus infections. NS1 has much to offer to flavivirus vaccine development.

## Figures and Tables

**Table 1:** List of anti-NS1 monoclonal antibodies, their virus specificity, and recognized epitopes, if known.

*Monoclonal* *Antibodies*	Antigenic Specificity ^1,2,3,4,5,6^	Epitope (NS1 Residues)	Reference
*2B7*	DENV complex		[52]
*3D1.4*	DENV complex	111–121	[53]
*1A12.3*	DENV complex	111–121	[53]
*4H3.4*	DENV complex	111–121	[53]
*3A5.4*	DENV complex	111–121	[53]
*33D2*	DENV complex	109–122	[14]
*19–5*	DENV complex	109–122	[14]
*DB16-1*	DENV complex		[54]
*DB20-6*	DENV complex	115–120	[54]
*DB29-1*	DENV complex	115–120	[54]
*15F3-1*	DENV1	110–117	[55]
*4F6*	DENV2	25–38	[56]
*4H2*	DENV2	127–143	[56]
*DB6-1*	DENV2		[54]
*DB12-3*	DENV2		[54]
*DB38-1*	DENV2		[54]
*DB41-2*	DENV2 & DENV4		[54]
*5H5.4*	DENV2 & DENV4	299–309	[53]
*1G5.3*	DENV2 & DENV4	299–309	[53,57]
*1H6*	JEV	146–150	[58]
*10F7*	JEV	1–16	[59]
*8B5*	JEV	65–80	[59]
*7G1*	JEV	249–264	[59]
*7C11*	JEV	265–280	[59]
*4E3*	JEV	265–280	[59]
*HA12*	JEV	265–280	[59]
*3H11*	JEV	337–352	[59]
*3G11*	JEV	337–352	[59]
*4E8*	JEV	337–352	[59]
*5C6*	JEV	337–352	[59]
*6D8*	JEV	337–352	[59]
*2B8*	JEV	225–233	[60]
*1E8*	JEV serocomplex	227–232	[59]
*4D1*	JEV serocomplex	925–934	[61]
*22NS1*	JEV serocomplex	172–352	[58]
*20B4*	TBEV	269–333	[62]
*29G9*	TBEV	1–33	[62]
*3G2*	Tembusu virus	269–274	[63]
*3C7*	WNV	895–901	[61]
*10NS1*	WNV	1–157 & 158–235	[64]
*14NS1*	WNV	236–352	[64]
*16NS1*	WNV	1–157	[64]
*17NS1*	WNV	1–157 & 236–352	[64]
*428, 423, 992, 917, 999, 979, 871, 925*	YFV & Alfuy virus		[51]
*Z11*	ZIKV	102	[65]
*Z15*	ZIKV	146	[65]
*Z18*	ZIKV	102	[65]
*Z17*	ZIKV	289 & 338	[65]
*749-A4*	ZIKV	289 & 338	[65]
*ZIKV-292*	ZIKV	101 & 177–178	[65]
*ZIKV-231*	ZIKV	265 & 314	[65]
*2H5*	ZIKV & DENV2	193–209	[56]
*4H1BC*	ZIKV & DENV2	193–209	[56]

^1^ DENV: dengue virus; ^2^ JEV: Japanese encephalitis virus; ^3^ TBEV: tick-borne encephalitis virus; ^4^ WNV: West Nile virus; ^5^ YFV: yellow fever virus; ^6^ ZIKV: Zika virus.

**Table 2:** Known roles of NS1.

Replication	Immune Response	Pathogenesis
Formation of replication complex	Suppress IFN-β induction	Promotes vascular leakage and platelet aggregation in DENV infection
Virus assembly	Promote complement-mediated cytolysis	Contributes to hyperpermeability in umbilical vein and brain endothelial cells in ZIKV infection
Virus maturation	Promotes antibody-dependent cellular cytotoxicity (ADCC)	YFV infects liver
	Antibody-dependent cellular phagocytosis (ADCP)	JE serocomplex viruses are neuroinvasive
